# An Exploratory Study of the Associations Between Epstein–Barr Virus Antibodies and Forgiveness Among Recipients of Relational Transgressions in the USA

**DOI:** 10.1007/s10943-024-02184-4

**Published:** 2024-11-30

**Authors:** John P. Crowley, Amanda Denes, Adam Richards, Joseph Whitt, Shana Makos

**Affiliations:** 1 University of Delaware, 250 Pearson Hall, Newark, DE 19716 USA; 2 University of Connecticut, Storrs, CT 06268 USA; 3 Furman University Greenville, Greenville, SC 29613 USA; 4 Wayne State College, Wayne, NE 68787 USA; 5 University of Illinois Urbana Champaign, Urbana, IL 61801 USA

**Keywords:** Forgiveness, Unforgiveness, Stress and coping, Immune health, Epstein–Barr virus, Religion, Investments

## Abstract

Forgiveness is an important component of many of the world’s religions that also has benefits for individuals’ health and relationships. Research on the health benefits of forgiveness is couched predominately in the stress and coping framework, which views forgiveness as buffering the stress associated with unforgiving feelings. This exploratory study (*N* = 47) elaborated on the stress and coping framework by investigating it in conversation with an evolutionary approach. Specifically, this study examined one’s own forgiveness index (i.e., the interaction of exploitation risk and relationship value) as moderating an association between forgiveness and Epstein–Barr virus (EBV) antibodies. The results indicated that forgiveness shared no significant association with EBV antibodies at low (16th percentile) levels of relationship value (*b* = − 11, *p* = .643), but shared an increasingly significant negative association at moderate (50th percentile: *b* = − 49, *p* = .038) and high (84th percentile: *b* = − 84, *p* = .009) levels, suggesting that forgiveness was more strongly linked to enhanced immune function when occurring in higher valued relationships. Implications for religion, theory, and methodological comparison are discussed.

## Introduction

Substantial evidence exists to support the health benefits of religiosity (Hall et al., 2008). One of the possible reasons for why religiosity may confer benefits in close relationships is due to the ubiquity of forgiveness as a concern across the world’s religions (McCullough & Worthington, 1999; McCullough et al., 2005). Put simply, the health benefits of religiosity may be a result of the presence of forgiveness in religious doctrine, encouraging people to repair relationships with those who have transgressed against them. Indeed, prior work has identified forgiveness as a mediator between religiosity and numerous positive outcomes. For example, such work found that higher religiosity was associated with greater forgiveness, which in turn predicted less distress (Olawa et al., 2022), greater post-traumatic growth (Lee & Kim, 2021), and greater meaning in life (Wnuk & Charzyńska, 2023). As such, investigating how forgiveness impacts health has implications for religion, as it can shed light on a behavior often endorsed by religions that impacts individuals’ health and well-being.

According to the stress and coping framework (Worthington & Scherer, 2004), which was born out of positive psychology, forgiveness is an emotion-focused coping strategy that helps to attenuate the negative effects of transgressions on stress-regulating systems in the body. Indeed, from a positive psychology perspective, forgiveness is understood as a character strength (Park & Peterson, 2008). Forgiveness is shown to encourage adaptive physiological responses in biological markers of cardiovascular, sympathetic nervous system, and hypothalamus–pituitary–adrenal axis (HPA) activity (see Crowley & Allred, 2020, for a review). Importantly, however, meta-analyses (Rasmussen et al., 2019) demonstrate *nonsignificant* associations between forgiveness and a host of physical health markers, including cortisol, infections, and bodily pain, with the exception being significant associations for markers relating to cardiovascular functioning. These mixed findings underscore a need for research to examine explanatory mechanisms for the forgiveness-health link and offer elaboration on the stress and coping framework.

The present study builds upon prior forgiveness-health research by seeking to combine the stress and coping framework with an evolutionary perspective that also accounts for the health benefits of forgiveness. Evolutionary models position religion as a signaling system that functions to encourage problem solving and group cooperation (Bulbulia & Sosis, 2011). Social relationships play a fundamental role in health and well-being (Lieberman, 2007) and, due to the importance of forgiveness in helping to maintain such relationships, its role in organized religions may serve as an evolutionary signal to aid in relational maintenance (Van Slyke and Szocik, 2020).

In the present study, we explored whether the relationship between forgiving responses to a romantic relational transgression and immunological health outcomes is dependent upon estimations of a forgiveness index, which signals the presence of a viable romantic relationship. Forgiveness and levels of antibodies to Epstein–Barr virus (EBV) were assessed for individuals who were (at the time of the study) negotiating the aftermath of a romantic relational transgression and had yet to forgive their partner. Such exploratory research is necessary to validate the links between forgiveness and health as individuals are actively managing severe relational issues, rather than relying solely on retrospective accounts after forgiveness has already occurred and the transgression may have been reframed in light of granting forgiveness. Taken together, this exploratory study contributes to research linking forgiveness to physical health and ultimately aids in understanding an important cognitive motivation that has been associated with religiosity and health.

## A Stress and Coping Perspective on Forgiveness and Health

Relational transgressions are common in romantic relationships (Blow & Hartnett, 2005). A relational transgression occurs when a romantic partner breaks an implicit or explicit relationship rule (Afifi et al., 2001). Relational transgressions are commonly cited as reasons for divorce in the United States (Scott et al., 2013).

Transgressions can pose threats to the health of recipients and perpetrators (Rasmussen et al., 2019), which may be due to feelings of unforgiveness that exist in the wake of transgressions (Worthington & Scherer, 2004). Unforgiveness is a term that describes the resentful and bitter feelings that emerge after relational transgressions and is characterized by grudge holding and rumination (Worthington & Scherer, 2004). Although unforgiveness refers to the unresolved negative emotions that result from experiences with transgressions, a reduction of unforgiveness does not imply the occurrence of forgiveness. As a psychological construct, *cognitive forgiveness* is conceptualized as reduced motivation to (a) exact revenge upon and (b) avoid the offender, as well as heightened motivation to (c) wish good will to the offender (McCullough et al., 2006). Forgiveness therefore requires the introduction of positive responses, such as benevolent motivations. As such, one can lessen their unforgiveness without fully forgiving the transgressor. Put differently, one can want less vengeance and avoidance for their offender, but forgiveness would occur only if they also experienced benevolence toward them.

Despite forgiveness being articulated in numerous religious and philosophical texts as something that is good for individuals (McCullough & Worthington, 1999), scholars have only recently sought to investigate its connection to physiological health. When unforgiving feelings are unmitigated, their impact is reflected not only in individuals’ psychosocial well-being, but also in their physiological and physical health (Cheadle & Toussaint, 2015; Crowley & Allred, 2020; Toussaint et al., 2020). Evidence suggests that unforgiving feelings are linked with hyperarousal in stress-regulating biological systems (Larkin et al., 2015). According to the stress and coping framework, forgiveness is likely to benefit immune system functioning through both direct mechanisms (e.g., at the cellular level and neuro-endocrine level, or through the release of antibodies) and indirect mechanisms (e.g., religion and social support; Worthington & Scherer, 2004). Despite such claims, few studies have sought to investigate associations between forgiveness and immune health and those have present mixed evidence for such a link. Two studies, that we are aware of, have investigated the relationship between immune health and forgiveness.

Seybold et al. (2001) discovered that greater forgiveness was associated with lower white blood cell counts and lower hematocrit levels (i.e., red blood cell percentage). An increase in white blood cells is a marker of infections (Brodin & Davis, 2017), as is an  increase in hematocrit levels (Moolenaar et al., 1995). Moreover, Owen et al. (2011; as cited in Larkin et al., 2015,1) identified higher CD4 cell (a type of white blood cell) percentage among individuals with HIV/AIDS who reported more forgiveness. Although these findings identify potential connections between immune health and forgiveness, scholars have appropriately called for additional research on the immune health implications of forgiving responses (Crowley & Allred, 2020; Lavelock et al., 2015).

## An Evolutionary Perspectives on Forgiveness and Health

In addition to the stress and coping framework, another predominant theoretical perspective for understanding forgiveness is the evolutionary approach (see Davis et al., 2015; Worthington et al., 2019). Evolutionary mechanisms can aid in understanding why forgiveness may benefit health. Humans are social animals and depend upon others and the resources provided by relationships to survive (Fitness & Peterson, 2008). It follows that “the successful maintenance of relationships requires forgiveness” (Fitness & Peterson, 2008, p. 8), and forgiveness has been identified as a threat-mitigating relational maintenance strategy (Ogolsky et al., 2017) that helps keep pair bonds intact. Forgiveness encourages self-sacrifice to reduce motivations toward avoidance and revenge, and this selfless quality of forgiveness likely also serves a superordinate goal toward accomplishing social harmony (i.e., reciprocal altruism, or cooperative management in relationships that benefit both self and other).

In evolutionary terms, the central function of forgiveness is that it promotes survival and reproduction through relationship repair, thereby preserving the benefits that social and romantic relationships offer for viability or fitness (Tooby & Cosmides, 2020). Forgiveness is also shown to regulate the body’s stress response (e.g., forgiving imagery encourages lower heart rate and mean arterial pressure compared to unforgiving imagery; Witvliet et al., 2001) and promote relaxation (e.g., imagining granting forgiveness encourages lower levels of tension measured through eye muscle activity; Witvliet et al., 2008). It follows that if it “feels good” physiologically to forgive (and confers physiological benefits), then it would be because forgiveness is instrumental in relational maintenance and thus contributes to basic evolutionary goals of survival and reproduction. As such, it is necessary to consider relational factors that are linked to superordinate evolutionary goals and their impact on the forgiveness process. Research connecting forgiveness with religion through an evolutionary lens, specifically, offers specificity surrounding how the decisions of forgiveness may confer health due to estimations of costs and benefits for fitness.

## The Evolutionary Function of Forgiveness as a Signal-Detection Task

It is theorized that the ubiquity of forgiveness across teachings of religions is an evolved mechanisms supporting survival of the human species (Gold & Davis, 2005). Indeed, scholars posit “that forgiveness is important in religious teachings because with forgiveness comes reconciliation, and thus the continuance of a community and the survival benefits that a community brings” (Gold & Davis, 2005, p. 122). More specifically, religion itself is conceptualized as a signaling system that is evolutionarily adaptive through its promoting of problem-solving cooperation (Bulbulia & Sosis, 2011) and forgiveness may be key to accomplishing such outcomes (McCullough et al., 2005), particularly in its role as a signal-detection task (Tan et al., 2017). Indeed, prior work has identified forgiveness as an explanatory mechanism of the positive effect of religiosity on health outcomes (Lawler-Row, 2010).

Couched within a signal detection theory (Green & Swets, 1966), a signal-detection task occurs in uncertain environments in which cost–benefit tradeoffs are estimated and result in biased cognitions that encourage behaviors that reflect the less costly error on fitness (Tan et al., 2017). Put simply, considering forgiveness allows recipients to assess the viability of a relationships after a perceived harmdoing. As Tan et al. (2017) suggest, “Forgiving is adaptive if a relationship with the ‘harmdoer’ will be fitness enhancing and not adaptive if the relationship will be fitness reducing, and the decision should be biased toward lowering the likelihood of the more costly error, which depending on the context may be either erroneously not forgiving or forgiving” (p. 27). An evolutionary approach therefore considers not just the costs and rewards of forgiveness, but from the perspective of superordinate goals and motivations related to resource management and survival.

If forgiveness is a signal-detection task, then it may be that forgiveness benefits a recipient’s immunological health when it is directed at reducing costly relational errors (e.g., forgiving someone who should not be forgiven; Tan et al., 2017). This possibility is explored below and specifically in relation to the VCA IgG antibodies to the Epstein–Barr virus (EBV) for recipients of relational transgression.

## Epstein–Barr Virus

We focused on a specific marker of immunological health—VCA IgG antibodies to EBV (henceforth, referred to simply as EBV antibodies)—in a sample of individuals who recently experienced a relational transgression in their dating relationship. Antibodies were investigated because evidence indicates that, for individuals who are seropositive for EBV (i.e., those with a blood test demonstrating the presence of the virus), viral infection symptoms are associated with higher levels of EBV antibodies (Nystad & Myrmel, 2007). EBV is a human herpesvirus that infects a majority of the population, with some estimates claiming that 80–90% of adults are infected with the virus by the age of 40 (Jones & Straus, 1987). Once an individual has contracted EBV, it remains in the body for life, existing in a dormant state and typically asymptomatic (Tao et al., 2006). When an individual’s immune system is weakened, EBV can become re-activated and release viral antigens into the bloodstream, thereby prompting the immune system to respond by releasing antibodies (Glaser et al., 1991). Given that EBV activation is linked to individuals’ exposure to chronic stress (Cacioppo et al., 1998), and relational transgressions are stressful for recipients to experience (McCullough et al., 2007), it is important to investigate whether behaviors or attitudes in the aftermath of transgressions are at all protective in limiting the reactivation of EBV.

## The Moderating Effect of the Forgiveness Index

Research on forgiveness from an evolutionary perspective has argued that, before a person decides to forgive an offender, an evolved forgiveness system estimates factors such as *exploitation risk* and *relationship value* (Burnette et al., 2012). Exploitation risk refers to the likelihood that a recipient will be a recipient of future harms. Relationship value refers to the viability of a relationship’s future and the potential for a recipient to receive rewards should they decide to remain within a relationship and reestablish or strengthen severed bonds with their partner (see Davis et al., 2015, for a review). Scholars argue that these two factors—exploitation risk and relationship value—constitute a person’s *forgiveness index* (Petersen et al., 2010). That is, when exploitation risk is low and relationship value is high, then the forgiveness index is higher. Although prior work has examined the forgiveness index exclusively as a predictor of forgiveness (Billingsley et al., 2023; McCullough et al., 2014; Tan et al., 2017), it is possible that the forgiveness index is implicated in whether forgiveness begets healthy outcomes for forgivers. Indeed, research has called for work to explore the possibility that health consequences of forgiveness depend on exploitation risk and relationship value (Davis et al., 2015).

Because this study focused on individuals that experienced severe relational transgressions, and some individuals had decided to remain within their relationship (at the inception of the study), it could be argued that a healthier and more evolutionarily adaptive response would be to seek revenge and/or to avoid offenders (i.e., remain in a state of unforgiveness). Put differently, severe transgressions may signal that the relationship will not be viable long-term (and thus will fail to contribute to superordinate goals for procreation and survival) and therefore, from an evolutionary perspective, individuals would benefit from *not* forgiving their partner’s offenses. If a function of forgiveness is to help repair [viable] relationships, then forgiveness may help alleviate stress (and be protective immunologically) when the forgiveness index is higher (i.e., exploitation risk is lower and relationship value is higher). However, when the forgiveness index is lower (i.e., exploitation risk is higher and relationship value is lower), it may suggest that the relational context is less evolutionarily viable and thus forgiveness under these circumstances may interrupt and/or counter its typical role in alleviating stress activation. In other words, when the risk of exploitation is high and the relationship has low value, it may be more protective to individuals’ health and well-being for them *not* to forgive. Therefore, a moderation model addressing the following hypothesis was tested:

H: An individual’s forgiveness index (i.e., relationship value and exploitation risk) moderates the association between forgiveness and EBV antibodies, such that forgiveness is negatively related to EBV antibodies when relationship value is higher and exploitation risk is lower, and positively related to EBV antibodies when relationship value is lower and exploitation risk is higher.

## Method

### Recruitment and Screening Procedures

Interested participants were recruited from classes at a large public university and were directed to a screening survey that assessed individuals’ eligibility to participate in the study. The study received approval from the institutional review board at the first author’s university on 5/21/2019. The screening survey ensured that participants were legal adults who (a) had experienced a relational transgression in a current romantic relationship within the previous six months and (b) considered the transgression to be at least moderately severe (i.e., rated at least 4 or above on a 9-point Likert scale). If participants met these inclusion criteria, they were then scheduled for a lab appointment and, upon completion of the study, compensated with extra credit for a class.

### Sample

In total, 47 participants were enrolled in the study.^2^ Participants ranged in age from 19 to 35 years (*M* = 21.5; *SD* = 0.35). Table 1 includes the participants’ demographic characteristics.

**Table 1:** Participants’ demographic characteristics (N = 47)

*Biological Sex*	*N*	*Percentage*
Female	37	78.7%
Male	10	21.3%
*Race*		
Asian/Pacific Islander	26	55.3%
White	11	23.4%
Hispanic/Latino	4	8.5%
Black/African American	3	6.4%
Races not specified	3	6.4%
*Sexual Orientation*		
Heterosexual	29	61.7%
Bisexual	13	27.7%
Gay	2	4.3%
Sexual Orientation not specified	3	6.4%
*Socioeconomic Status*		
Poor	1	2.1%
Working Class	9	19.1%
Lower Middle Class	9	19.1%
Upper Middle Class	23	48.9%
Upper Class	5	10.6%

### Study Procedure

Upon arrival to the lab, participants were greeted and guided through the informed consent process by a lab attendant. Once consent was gathered, blood samples were collected using the dried blood spot (DBS) procedure, which involved several steps. First, participants were instructed to use a micro lancet to prick their index finger. Second, participants produced five (and no less than three) blood droplets on a protein saver card, which contained filter paper. Third, a lab attendant stored the protein saver card for four hours to allow the blood droplets to dry completely. Fourth, the lab attendant then stored the protein saver card in a -20 Celsius freezer. After participants provided the sample, they were instructed to complete a survey that included assessments about relational value and exploitation risk, relational commitment, and forgiveness. Participants also completed items relating to the transgression.

## Measures

### EBV Antibodies Assay Method

Antibodies to Epstein–Barr viral capsid antigen (EBV VCA IgG) were measured using a commercial ELISA kit supplied by IBL International (Hamburg, Germany; IBL catalog # RE57351) that allows for quantification of the antibody in arbitrary units. All specimens were run in duplicate. The kit manufacturer reports that the assay shows no cross-reactivity with antibodies to other common viruses tested (parvovirus B19, VZV, HSV 1, CMV, measles, mumps, toxoplasmosis, and rubella) and that the assay demonstrated linearity (recovered values 85%—111% of the expected values). Precision tests for high (68 U/mL), medium (23 U/mL), and low (9 U/mL) levels resulted in intra-assay coefficients of variation (CVs) of 7%, 6%, and 9%, respectively, and inter-assay CVs of 19%, 8%, and 18%, respectively. Based on comparison to the kit manufacturer's suggested cutoff control (20 U/mL), all samples were strongly positive for anti-EBV antibody, indicating that all participants were seropositive for EBV.

### Forgiveness

Forgiveness was assessed with the 18-item Transgression-Related Interpersonal Motivations Scale (TRIM; McCullough et al., 1998). Items were anchored from 1 = *strongly agree* to 5 = *strongly disagree* and assessed participant motivations to avoid (e.g., “I keep as much distance between us as possible”), seek revenge (e.g., “I’ll make him/her pay”), or act benevolently (e.g., “I find it difficult to act warmly toward him/her”) toward their romantic partner. Strong measurement reliability was found for the total TRIM scale (α = 0.90).

### Exploitation Risk

Exploitation risk was measured with five items (Burnette et al., 2012) anchored from 1 = *completely disagree* to 5 = *completely agree.* The items included statements such as “I feel threatened by him/her,” “I feel like he/she might do something bad to me again,” and “I can’t predict how he/she is going to treat me in the future.” The items were demonstrably reliable in measuring the construct (α = 0.77).

### Relationship Value

Relationship value was measured with five items (Burnette et al., 2012) anchored from 1 = *completely disagree* to 5 = *completely agree.* The items included statements such as “Our relationship is very rewarding to me,” “He/she still plays a key role in my life,” and “I feel like our interests and personalities are very compatible.” The items were demonstrably reliable in measuring the construct (*α* = 0.88).

## Results

Initial data analysis involved inspection for deviations from normality for the primary independent and dependent variables. No significant skewness or kurtosis among the measured variables emerged, and therefore, no transformations were performed (skewness and kurtosis (< 3 and < 10, Kline, 1998). Bivariate correlations between the primary variables were then assessed for evidence of multicollinearity. There were no problematic relationships among the primary variables in the analyses (all VIF values ≤ 2.43). Correlations among the primary variables are depicted in Table 2.

**Table 2:** Correlations among Primary Variables

	*M*	*SD*	1	2	3	4	5
1. EBV Antibodies	426,213.64	38,954.31	–				
2. Forgiveness	3.61	.71	− 13	–			
3. Relationship Value	3.86	.97	− 15	.71**	–		
4. Exploitation Risk	2.84	.88	.06	− 28	− 15		
5. Relationship Status	1.17	.38	.30*	− 34*	.42**	.17	
6. Age	21.49	2.4	.30*	.25	.20	− 07	− 09

*N* = 47. *Relationship* s*tatus* reflects whether participant identified still being in the relationship in which the transgression occurred at the time of the study (0 = yes, 1 = no). This variable was coded where the higher number indicated that the participant was no longer within the relationship

*p < .05. ** *p* < .01. *** *p* < .001

### Preliminary Analyses

Participants reported the severity of the transgression on a single item measure, anchored from 1 = *not at all severe* to 7 = *extremely severe*, and indicated that, on average, the transgressions were moderately severe (*M* = 5.21, *SD* = 1.02). With respect to time, the majority of the transgressions occurred within 0–1 months prior of the study (*n* = 13, 27.7%), and then also within 2–3 months (*n* = 11, 23.4%), 1–2 months (*n* = 8, 17.0%), and 5–6 months prior (*n* = 8, 17.0%) of the study. Others reported experiencing their transgression 3–4 months (*n* = 4, 8.5%) and 4–5 months (*n* = 3, 6.4%) prior to the study. Qualitative descriptions of transgressions were coded using Roloff et al.’s (2001) categories. Participants reported experiencing a lack of openness/honesty (*n* = 18, 34.0%), insensitive behavior (*n* = 14, 29.8%), romantic infidelity (*n* = 7, 12.8%), and dominating behavior (*n* = 4, 8.5%), and feeling that their relationship was of low priority (*n* = 4, 8.5%).

Possible covariates to include in the primary analyses were explored based on their connections to EBV. Sex and age were considered given prior research identifying differences between males and females in EBV (Sasaki et al., 2020) and in seropositivity for EBV with increases in age (Kuri et al., 2020). An independent samples *t* test with equal variances not assumed demonstrated no significant difference (*t*[45] = -0.33, *p* > 0.05) between males’ (*M* = 38,944.24, *SD* = 47,582.36) and females’ (*M* = 43,618.07, *SD* = 36,988.54) EBV antibody levels. Age was significantly positively correlated with EBV antibody level (*r* = 0.30, *p* < 0.05).

Covariates that might influence perceptions of the offender (i.e., exploitation risk), the romantic relationship (i.e., relationship value), or motivations to forgive were also considered. These included whether individuals had remained within the relationship (at the time of their participation) after experiencing the transgression (hereafter referred to as relationship status), the time that had lapsed since experiencing the transgression, and biological sex. In total, 39 participants remained in the relationship with the partner who committed the transgression and eight were no longer within the relationship. An independent samples *t* test with equal variances not assumed demonstrated a significant difference in relationship value (*t*(45*)* = 2.656, *p* = 0.01), with those who remained in the relationship (*M* = 4.04, *SD* = 0.86) valuing the relationship more than those who were no longer in the relationship (*M* = 2.98, *SD* = 1.06). Similar results emerged with forgiveness (*t*(45*)* = 2.73, *p* = 0.01), with those who remained in the relationship (*M* = 3.72, *SD* = 0.69) forgiving more than those who were no longer in the relationship (*M* = 3.08, *SD* = 0.58). With respect to sex differences, an independent samples *t* test with equal variances not assumed indicated sex differences (*t*(45*)* = 1.76, *p* = 0.04) in reports of relationship value, with males (*n* = 10; *M* = 4.18; *SD* = 0.48) reporting greater relationship value than females (*n* = 37; *M* = 3.77, *SD* = 1.05). No other variables were significantly different based on biological sex or relationship status. Moreover, the time that had lapsed since the transgression was not significantly correlated with any of the primary variables in this study (*p* > 0.05). Therefore, age, sex, and relationship status were included as covariates in the primary analyses. All variables were standardized.

### Hypothesis Testing

We predicted that forgiveness would have a stronger negative association with EBV antibodies at increasing levels of relationship value and decreasing levels of exploitation risk. We tested this prediction using PROCESS 3.5 (Model 3, 95% CIs; Hayes, 2018a, 2018b) in which EBV antibodies were regressed onto forgiveness, relationship value, and exploitation risk, along with their interactions, while controlling for biological sex (0 = female, male = 1), relationship status (0 = in relationship, 1 = not in relationship), and age. As demonstrated in Table 3, the regression analyses (*R*^2^ = 0.44, *F*[10, 36] = 2.86, *p* = 0.01)^3^ revealed a significant two-way interaction effect between forgiveness and relationship value (*b* = -0.43, *p* = 0.019), and an main effect that was trending toward statistical significance for forgiveness (*b* = − 0.43, *p* = 0.061), as well as a two-way interaction effect that was trending toward statistical significance between forgiveness and exploitation value (*b* = − 0.44, *p* = 0.052). All covariates were significant: EBV antibodies were higher in males compared to females (*b* = 0.35, *p* = 0.029), in those not in the relationship compared to those still in the relationship (*b* = 0.31, *p* = 0.032), and in older people (*b* = 0.46, *p* = 0.002). However, there was no significant three-way interaction between forgiveness, relationship value, and exploitation risk (*b* = − 0.06, *p* = 0.785); as such, the hypothesis was not supported.

**Table 3:** Ordinary Least Squares Regression Coefficients (With Standard Errors [SEs]) for Moderation Model Predicting EBV Antibodies (*N* = 47)

	*b (SE)*	95% CIs	
		*LL*	*UL*
Constant	0.19 (0.15)	− 0.12	0.50
Forgiveness	− 0.43 (0.22)^*†*^	− 0.88	0.02
Relationship Value	− 0.08 (0.22)	− 0.52	0.37
Exploitation Risk	− 0.28 (0.17)	− 0.62	0.06
Forgiveness × Relationship Value	− 0.43 (0.17)*	− 0.78	− 0.07
Forgiveness × Exploitation Risk	− 0.44 (0.22)^*†*^	− 0.88	0.00
Relationship Value × Exploitation Risk	0.27 (0.20)	− 0.14	0.68
Forgiveness × Relationship Value × Exploitation Risk	− 0.06 (0.22)	− 0.50	0.38
Cov: Sex	0.35 (0.15)*	0.04	0.65
Cov: Age	0.46 (0.13)**	0.18	0.74
Cov: Relationship Status	0.31 (0.14)*	0.03	0.60

Sex was coded so 0 = *female* and 1 = *male*. Relationship status was coded so 0 = *in relationship* and 0 = *not in relationship*

^*†*^*p* ≤ .06. *p < .05. ** *p* < .01

**Fig. 1:**
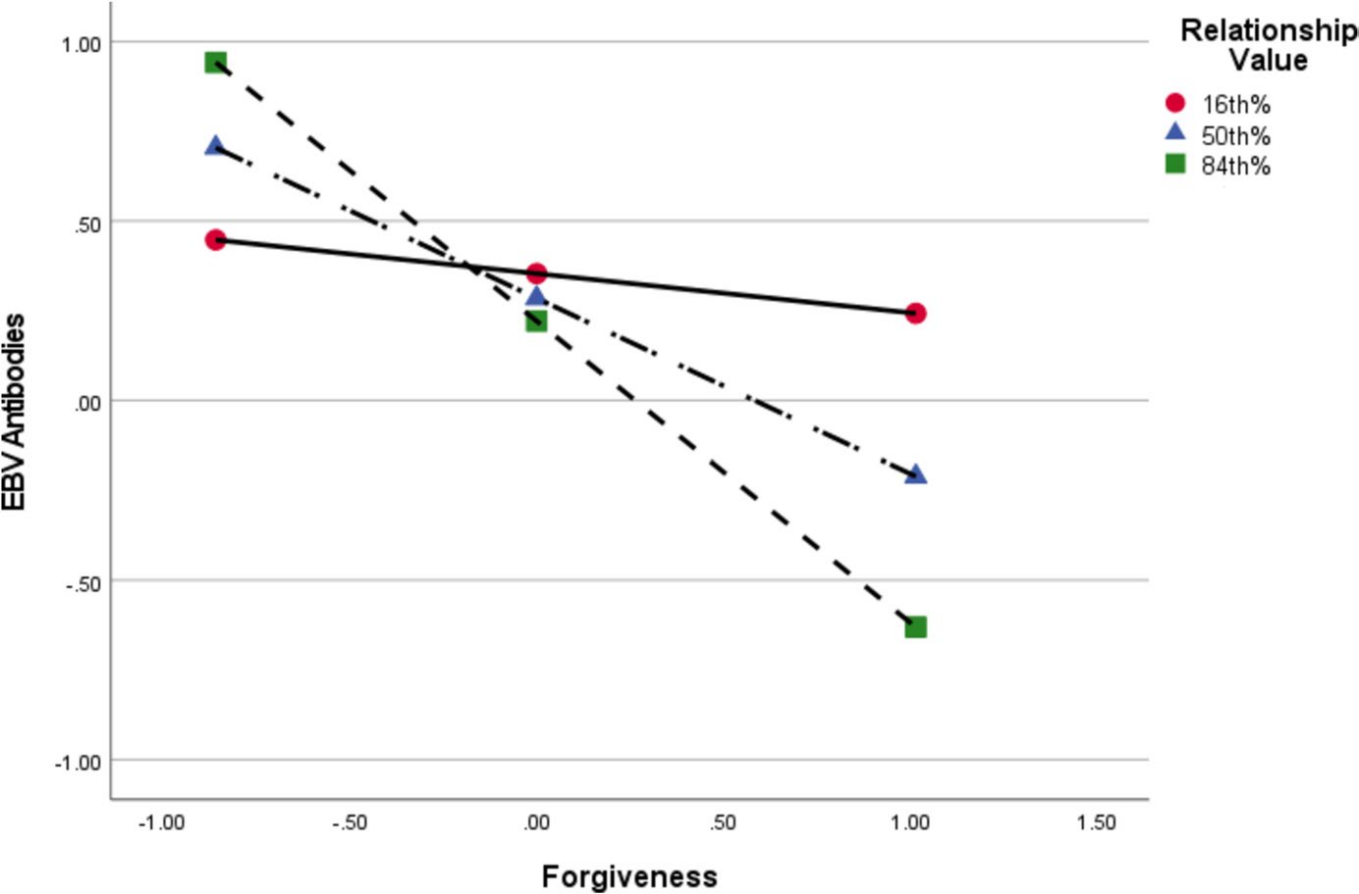
Conditional Effects of Forgiveness on EBV Antibodies at Levels of Relationship Value. Values reflect a standardized measures. Relationship value refers to one’s own perception of the viability of their relationship’s future. It represents assessments of rewards received should they decide to remain within their relationship and reestablish or strengthen severed bonds with their partner (see Davis et al., 2015, for a review)

We probed the conditional effects of the significant two-way interaction between forgiveness and relationship value. As shown in Fig. 1, forgiveness increasingly associated with reduced EBV antibodies as relationship value increased. Forgiveness had no significant effect on EBV antibodies at low (16th percentile) levels of relationship value (*b* = − 0.11, *p* = 0.643, 95%CI[− 0.58, 0.37]), but had an increasingly significant effect at moderate (50th percentile: *b* = -0.49, *p* = 0.038, 95%CI[− 0.95, − 0.03]) and high (84th percentile: *b* = − 0.84, *p* = 0.009, 95%CI[− 1.45, − 0.23]) levels. That is, forgiveness was more strongly linked to enhanced immune function when occurring in higher valued relationships.

**Fig. 2:**
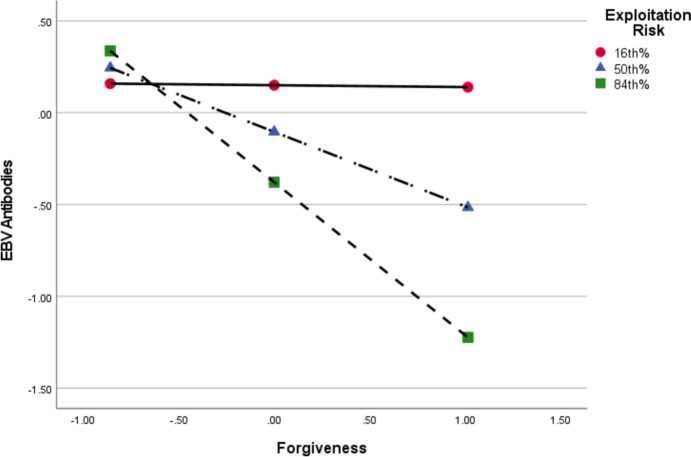
Conditional Effect of Forgiveness on EBV Antibodies at Levels of Exploitation Risk. Values reflect standardized measures

We engaged in an equivalent analysis for the two-way interaction that was trending toward statistical significance between forgiveness and exploitation risk. As shown in Fig. 2, forgiveness increasingly associated with reduced EBV antibodies as exploitation risk increased. Forgiveness had no significant effect on EBV antibodies a low (16th percentile) levels of exploitation risk (*b* = − 0.01, *p* = 0.964, 95%CI[− 0.47, 0.45]), but had an effect that was trending toward statistical significance at moderate levels (50th percentile: *b* = − 0.41, *p* = 0.069, 95%CI[− 0.84, 0.03]) and a significant effect at high (84th percentile: *b* = − 0.83, *p* = 0.027, 95%CI[− 1.56, − 0.10]) levels. That is, forgiveness was more strongly linked to enhanced immune function when occurring in relationships having higher exploitation risk.

### Sensitivity Analyses

Given the exploratory nature of the study, we sought to test alternative models with fewer inputs to attenuate concerns regarding the possibility of Type II error given the small sample size. The present sample is adequately powered to detect a large effect, though insufficient evidence exists in prior research to suggest that a large effect can be expected among these relationships. These sensitivity analyses were therefore conducted in an effort to determine whether the interaction effect of EBV antibodies on relationship value persists in statistical models with inputs that are sufficient to determine moderate or small effects (see power analysis in Appendix). These models, which are fully detailed in Appendix 1, provided evidence that the interaction remained significant and proved to be robust across these simplified models.

## Discussion

In an effort to help clarify the forgiveness-health link and to specifically develop an understanding of the associations between forgiveness and immunological health, this exploratory study applied an evolutionary logic couched in the understanding that decisions about forgiveness reflect a signal-detection task concerning the viability of a relationship (Tan et al., 2017). We drew upon work positioning religion as a signaling system that serves an evolutionary function by its encouragement of cooperation and problem-solving via mandates like forgiveness (Bulbulia & Sosis, 2011). From this foundation, we examined whether the link between forgiveness and immune health is conditionally based on the forgiveness index; that is, forgiveness is likely to confer costs when the value of a relationship is low and the continued risk of further exploitation by the offender is high.

The findings do not support the forgiveness index in terms of the joint interaction of estimations of relationship value and exploitation risk in moderating the forgiveness-immunological health link. Instead, the results indicate that forgiveness is related to lower EBV antibodies for only those who highly value their relationship. Surprisingly, the same pattern of findings emerged for exploitation risk, whereby forgiveness was linked with lower EBV antibodies for those who reported *higher* (and not lower, as was hypothesized) exploitation risk. These findings and their implications are discussed below.

### Forgiveness is Immunoprotective for a Highly Valued Relationship

The results identify that, at high levels of relationship value, forgiveness was negatively linked with EBV antibodies. These results support prior conceptions of forgiveness as functioning to help relationships repair (Ogolsky et al., 2017; Rusbult et al., 2005) but qualifies that forgiveness may not uniformly incur health benefits in doing so, as prior work has suggested (Worthington & Scherer, 2004). Rather, when forgiveness functions to repair relationships that are fitness-enhancing (i.e., when relationship value is reported at high levels), then it may incur less stress for recipients and exact less of an impact on immunological functioning. Alternatively, when cognitive forgiveness processes repair relationships that are perceived to be of a lower value, then forgiveness may not be meaningfully associated with EBV antibodies. Thus, the forgiveness-immune health link appears to be conditionally dependent upon one’s own perception of a highly valued relationship.

Unattenuated transgression-related stress likely hyperactivates biological systems that regulate the body’s stress response and increases one’s vulnerability to illness and disease through a taxed immune system. Whereas the findings suggest that forgiveness may be able to help regulate the stress response (for those who perceive their relationship to be of high value), forgiving may not be costly for those who deem the value of the relationship to be low. Thus, for romantic partners in lowly valued relationships, the presence of forgiveness, or lack thereof, may be unlikely to significantly protect or compromise their immune systems.

Although the findings do not support the forgiveness index, they may still support the view of forgiveness as serving an evolutionary function within religion. If forgiving a highly valued partner is the less costly error on fitness, then these data support, at least partially, the idea that forgiveness is a signal detection task (Billingsley et al., 2023; Tan et al., 2017) and its role in religion, as a signaling system (Bulbulia & Sosis, 2011), may enhance fitness through its promotion of problem-solving and cooperation. However, for those who forgive when signals would suggest costs to fitness (i.e., forgiving a lowly valued partner), the influence of forgiveness may not be harmful, but rather neutral in its impact, neither increasing nor decreasing the physiological harms of experiencing relational transgressions.

These findings may also help to clarify how forgiveness explains the relationship between religiosity and health. Forgiveness is positioned as an explanatory mechanism of the benefits of religiosity on health (Lawler-Row, 2010), but prior work has shown that religiosity can both encourage and discourage forgiveness depending on the recipients’ appraisal of the transgression (Tsang et al., 2020). It may be that the effect of religiosity on health through forgiveness is similarly conditionally dependent. That is, as we consider forgiveness in religion from an evolutionary perspective, it is important for future research to account for the relational and contextual factors that signal the viability of relationships for fitness, as such factors may moderate the mediated effect of religion on health outcomes through forgiveness.

Although two-way interaction effects emerged, the results failed to provide evidence of a significant three-way interaction between relationship value, exploitation risk, and forgiveness. The forgiveness index is postulated to reflect a *joint* interaction of recipients’ estimations of relationship value and exploitation risk (Tan et al., 2017), but the impact of such joint effects on the forgiveness-health link was not supported in this study. Moreover, whereas relationship value at high levels was a significant moderator, it did not significantly moderate the forgiveness-EBV link at lower levels. The impact of high relationship value on forgiveness and health aligns with prior work that has conceived of decisions to forgive as a signal-detection task (Billingsley et al., 2023; Tan et al., 2017) that, from an evolutionary perspective, supports individuals in estimations of the viability of a relationship.

This exploratory study took a novel approach and was responsive to calls to apply the forgiveness index to understand the health outcomes of forgiveness (Davis et al., 2015), ultimately offering the first application (to our knowledge) of the forgiveness index in this context. It is clarifying and instructive to future research that relationship value is a factor that may conditionally influence the extent to which forgiveness procures immunological health outcomes for forgivers when it is assessed at higher levels, but not also at lower levels. It may be that the forgiveness index operates in influencing health in ways that are different than how it is shown to promote conciliatory responses like forgiveness (e.g., Petersen et al., 2010).

### Forgiveness is Immunoprotective When Exploitation Risk is Salient

Although the moderating effect of exploitation risk was approaching significance (*p* < 0.06), due to the small sample size and the subsequent constraints on statistical power to detect differences, the interaction was probed further to examine the levels of exploitation risk at which forgiveness was associated with EBV antibodies. The results surprisingly indicate that forgiveness was more strongly linked to lower EBV antibodies when occurring in relationships having higher exploitation risk. Importantly, however, the average self-report of exploitation risk was below the scale’s midpoint (*M* = 2.84; *SD* = 0.88) and approximately 23% of the sample reported at 2.0 or below on the scale (with only 6 participants or 11% of the sample reporting scores from 4.0 to 4.6). As such, most participants were not concerned that their partner would exploit them in future. Many of the participants who reflect the designation of having “high exploitation risk” would be captured somewhere in between the “neither disagree or agree” to the “somewhat agree” scale point anchors (3.0—3.8; approximately 24% of the sample). Thus, exploitation risk that is designated as high in terms of this sample is more accurately reflecting what would best be considered moderate exploitation risk.

Although not originally theorized and counter to our predictions (as well as to the predictions of the forgiveness index, broadly), the hard-to-get phenomenon (Finkel & Eastwick, 2009) and sexual selection (Baker & Maner, 2009) may aid in understanding why it is that exploitation risk emerged as a significant moderator in this way. A moderate exploitation risk may be reflective of underlying assessments of attraction and risk-taking. Offenders who are either perceived to be not at all difficult to attract or who pose limited to no risk (or engage in little risk-taking behavior) may offer little in terms of fitness gains. In other words, these individuals may not be particularly desirable, and thus forgiveness is less advantageous because it does not serve to maintain a less viable partner. Alternatively, those who present the greatest risk for further exploitation would also reasonably reflect an unviability to the relationship context. It may be that some exploitation risk (but not so much that the recipient feels unsafe) is important for the viability of a relationship. Couched in this perspective, the choice to forgive with some exploitation risk may be a decision in favor of the less costly error on fitness and thus support the view of forgiveness as a signal detection task (Billingsley et al., 2023; Tan et al., 2017). Future research should more directly test accompanying evolutionary hypotheses, such as the hard-to-get phenomenon, to assess the costliness of the error of forgivingness on fitness when some exploitation risk is present.

It is also possible that, for some individuals, forgiveness represents a religious mandate and garners greater health benefits when it is granted *despite* the risk of future exploitation. For example, some religions promote forgiving one’s worst offenders (e.g., turn the other cheek)—individuals who would likely have a high exploitation risk. Thus, forgiveness of such individuals may be linked to better health outcomes because it might signal a highly religious person who is concurrently experiencing the known benefits of religiosity for health (Shattuck & Muehlenbein, 2020). These possibilities are speculative, and the extent to which exploitation risk is important—or of little consequences, as our findings suggest—for the viability of a relationship is something that future research should seek to disentangle further.

### Implications and Limitations

The present study conceptualized forgiving responses as functioning as an evolutionarily adaptive signal-detection task that may aid in encouraging viable relationships to repair in the aftermath of perceived wrongdoings (Ogolsky et al., 2017). Whereas the results fail to support the forgiveness index in terms of the joint interaction of relationship value and exploitation risk, both variables did emerge as being explanatory in terms of the forgiveness-immunological link. The results suggest that screening for relational dynamics prior to employing forgiveness promotion interventions is important, as failing to do so may have negative implications for forgiveness granters’ health. Specifically, future applied work should screen for exploitation risk and relationship value to assess the extent to which forgiveness is likely to benefit individuals or if existing levels of unforgiveness are signaling a relational context that should be attended to through different clinical or interventional methods (e.g., Buhagar, 2021). Indeed, Worthington and Scherer (2004) note that unforgiveness could “boost short-term immune responses” (p. 398) because it alerts individuals to the dangers present in their environment and that unforgiveness is perhaps only harmful to individuals in the long-term, but potentially beneficial in the short-term.

The current findings contribute in important ways to the research on forgiveness as an important behavior in many religions linked to health but are not without limitations. First, despite attempts to recruit from the community, the sample was predominately white, heterosexual, and college-aged, which limits the generalizability of the findings beyond this population. Second, although studies using physiological data have a lower measurement error and therefore warrant smaller sample sizes (Floyd & Afifi, 2011), the study would have nonetheless benefitted from a larger sample to rule out possible Type II errors and the potential for spurious findings that can accompany small sample sizes (Cao et al., 2024). At best, our sample is sufficiently powered to detect large effects, but there is no clear precedent for such an effects size based on prior research. Thus, caution should be applied to any conclusions drawn from these exploratory findings until future research is conducted to replicate them among larger samples of aggrieved relational partners. There is a possibility that a Type II error is present in the findings, particularly concerning the role of exploitation risk as either an independent or joint moderator (with relationship value). The findings for exploitation risk are counter to the theorized predictions of the forgiveness-index, and so these findings, in lieu of the small sample, should be interpreted circumspectly. The findings of the sensitivity analysis, however, further support the likely important role of relationship value in interacting with forgiveness to predict EBV antibodies for those negotiating the wake of relational transgressions. The findings of this exploratory study demonstrate a conditional effect of relationship value and exploitation risk on associations between forgiveness and EBV antibodies; however, the magnitude of this effect would need to be substantiated in future research with larger samples that allow for such estimations. In addition to recruiting a larger and more diverse sample, future research should seek to replicate these findings using a longitudinal design to determine how EBV antibodies might change as partners adapt to the new relational roles and routines that follow transgressions. One possibility is that forgiveness and EBV antibodies could be explained by the relational turbulence (see Solomon et al., 2016) that follows major transitional points for romantic partners. Moreover, future work should include measures assessing different motivations to forgive that more directly tap into the role of religion and/or religiosity in the forgiveness process, as well as its link to health. Indeed, it would be an important future direction to include measures of [religious] motivations to forgive that are not explicitly or implicitly linked to evolutionary explanations to see if they moderate or mediate the associations between forgiveness and health in ways that are distinct from what is shown in this study with the forgiveness index. Future research should also screen for the presence of mental health, as it is shown to influence levels of forgiveness (Toussaint et al., 2016), as well as physical health (particularly) stress given its role in influencing levels of EBV antibodies (Sausen et al., 2021). Moreover, it would be helpful to screen for any intervening methods of coping that have occurred for participants since their experience with the transgression such as with professional counseling or therapy (Akhtar & Barlow, 2018). It would also be important to consider the role of the communication of forgiveness in future work to see how, if at all, viable relationships beget greater, or different, forgiveness communications (see Waldron & Kelley, 2005). Beyond sample size, it is also possible that participants were subject to social desirability biases in their self-reports of levels of forgiveness, relationship value, and exploitation. Future work would benefit by accounting for such potential biases. Taken together, the extent to which forgiveness communication has implications for physical health outcomes, and whether the religious and relational context influences such connections, would be an important avenue for future research.

The findings of this study offer insights on the physiology of forgiveness and suggest that not only is the relationship between forgiveness and health complex, but that forgiveness may be health promoting, though perhaps only under certain conditions (i.e., when relationship value is high and exploitation risk is moderate). Such findings add important complexity to current understandings of the linkages between forgiveness and health and serve to move this line of research forward by specifying the conditions under which forgiveness may come with relational and physical health risks, as well as offering future directions for more directly linking religion to forgiveness and health. Though exploring the links between forgiveness and physiology is no longer uncharted territory (Witvliet et al., 2001), this line of research still requires considerable work to tease apart the complex relationship between forgiving motivations and physical health.

## Appendix 1: Sensitivity Analysis

A sensitivity analysis was conducted by assessing three additional models with varying inputs to determine whether the interaction between forgiveness and relationship value persisted in more parsimonious models. These three models included at most five variables each. This approach is supported by a power analysis that indicated that, in order to detect a moderate effect (f2 = 0.25) using an alpha of 0.10, power of 0.80, and with 5 total predictors, a sample size of 47 is required.

Our first model omitted the covariates but retained the main effects of forgiveness, relationship value, and exploitation risk, as well as the latter two variables’ separate interactions with forgiveness. According to this 5-predictor model, the interaction between forgiveness and relationship value remained significant (b = − 0.17, SE = 0.17, *p* = 0.015, 95%CI[− 0.63, − 0.07]). When decomposed using the Johnson-Neyman technique, the interaction indicated that forgiveness increasingly associated with reduced EBV antibodies for those particularly high in relationship value (transition point = 1.535, representing upper 6.24% of participants, b = − 0.66, *p* = 0.05, 95%CI[− 1.31, 0.0001]). This conditional effect replicates that which emerged in the results reported in the manuscript. Similarly, in the second model, we further reduced the number of predictors by omitting the interaction between forgiveness and exploitation risk, but continued to control for the main effect of exploitation risk. In this 4-predictor model, the interaction between forgiveness and relationship value was again significant (b = − 0.34, SE = 0.14, *p* = 0.016, 95%CI[− 0.62, − 0.07]). Finally, in a third model, we omitted the effect of exploitation risk, only accounting for the main effects of forgiveness and relationship value, as well as their interaction. Again, in this 3-predictor model, the interaction between forgiveness and relationship value was significant. (b = -0.29, SE = 0.12, *p* = 0.022, 95%CI[-0.54, -0.04]).

This sensitivity analysis was conducted to test more parsimonious models to check if the results replicate across the models, making them more trustworthy. As detailed above, the interaction in question remained significant and proved to be robust across models. Thus, we take this as evidence that a legitimate effect emerged in the context of an exploratory study. Please see Table

**Table 4:** Ordinary Least Squares Regression Coefficients (With Standard Errors [SEs]) for Moderation Models Predicting EBV Antibodies with Five, Four, and Three Predictors (N = 47)

	Five Predictors			Four Predictors			Three Predictors		
	b (SE)	95% CIs		b (SE)	95% CIs		b (SE)	95% CIs	
		LL	UL		LL	UL		LL	UL
Constant	0.21 (0.18)	− 0.14	0.57	0.24 (.17)	− 0.10	0.58	0.20 (0.16)	− 0.13	0.53
Forgiveness	− 0.12 (0.23)	− 0.57	0.34	− 0.07 (0.21)	− 0.49	0.35	− 0.02 (0.20)	− 0.43	0.38
Relationship Value	− 0.36 (0.23)	− 0.81	0.10	− 0.33 (0.22)	− 0.78	0.11	− 0.31 (0.22)	− 0.75	0.13
Forgiveness × Relationship Value	− 0.35 (0.14)*	− 0.63	− 0.07	− 0.34 (0.14)*	− 0.62	− 0.07	− 0.29 (0.12)*	− 0.54	− 0.04
Exploitation Risk	− 0.17 (0.17)	− 0.52	0.17	− 0.15 (0.17)	− 0.48	0.19			
Forgiveness × Exploitation Risk	− 0.12 (0.20)	− 0.52	0.29						

^*^*p* < 023. Model with five predictors included main effects of forgiveness, relationship value, and exploitation risk, as well as the latter two variables’ separate interactions with forgiveness. Model with four predictors included main effects of forgiveness, relationship value, and exploitation risk, but only the interaction between forgiveness and relationship value. Model with three predictors included the main effects of forgiveness and relationship value as well as their interaction, omitting the influence of exploitation risk altogether

4 for a detailed description of the sensitivity analysis results.

## Footnotes

1 The full text for Owen et al. (2011) could not be located.

2 Smaller sample sizes are common in psychophysiological research exploring interpersonal communication processes (e.g., Aloia & Solomon, 2015; Denes et al., 2020), as the lack of measurement error when employing physiological assessments is advantageous in terms of sample size requirements for achieving acceptable power (Floyd & Afifi, 2011).

3 Compared to the main effects model that excluded interactions (*R*^*2*^ = .24), this model accounted for a significant increase in explained variance (∆*R*^*2*^ = .20, ∆*F*[4, 26] = 3.30, *p* = .02).
